# The Equidae from Cooper’s D, an early Pleistocene fossil locality in Gauteng, South Africa

**DOI:** 10.7717/peerj.6909

**Published:** 2019-05-15

**Authors:** Shaw Badenhorst, Christine M. Steininger

**Affiliations:** 1 Evolutionary Studies Institute, University of the Witwatersrand, Johannesburg, South Africa; 2 DST-NRF Centre of Excellence in Palaeosciences, University of the Witwatersrand, Johannesburg, South Africa

**Keywords:** Equidae, *Eurygnathohippus cornelianus*, *Equus capensis*, Cooper’s D, Hipparion

## Abstract

Cooper’s D is a fossil locality in the Bloubank Valley close to other important sites such as Sterkfontein and Kromdraai in Gauteng, South Africa. The fossil deposits of Cooper’s D date to 1.38 ± 0.11 Ma. Hominins like *Paranthropus robustus* and early *Homo* have been recovered from Cooper’s Cave. We report here on the Equidae remains. Our sample contains specimens from the extinct *Equus capensis,* and a specimen which represents an extinct hipparion *Eurygnathohippus cf. cornelianus*. This particular specimen was previously identified as plains zebra (*Equus quagga*). The contribution of Equidae to the total fossil assemblage of Cooper’s D is relatively low, and these remains were likely accumulated by various predators such as spotted and brown hyenas and leopards. The Equidae, as well as the other fauna from Cooper’s D supports the existence of grassland, wooded and water components in the vicinity of the site.

## Introduction

The Bloubank Valley in the Cradle of Humankind close to Johannesburg in South Africa is well-known for the range of hominin taxa discovered at fossil localities like Sterkfontein, Kromdraai and Swartkrans dating from the Plio-Pleistocene. The animals found associated with these hominins are of considerable interest, and a large body of research has been produced, investigating aspects such as the palaeo-environments (e.g., Reed, 1996; Adams, 2006; Kuhn, Werdelin & Steininger, 2017; Sénégas & Thackeray, 2008; Steininger, 2011; Adams et al., 2016), indirect dating of deposits through faunal seriation (e.g., McKee, Thackeray & Berger, 1995; Badenhorst et al., 2011; Van Zyl, Badenhorst & Brink, 2016), taphonomy (e.g., Brain, 1981; Adams et al., 2007; Val, Taru & Steininger, 2014; Kruger & Badenhorst, 2018) and taxonomy (e.g., Churcher, 1970; Churcher, 1974; Vrbra, 1976).

**Figure 1:**
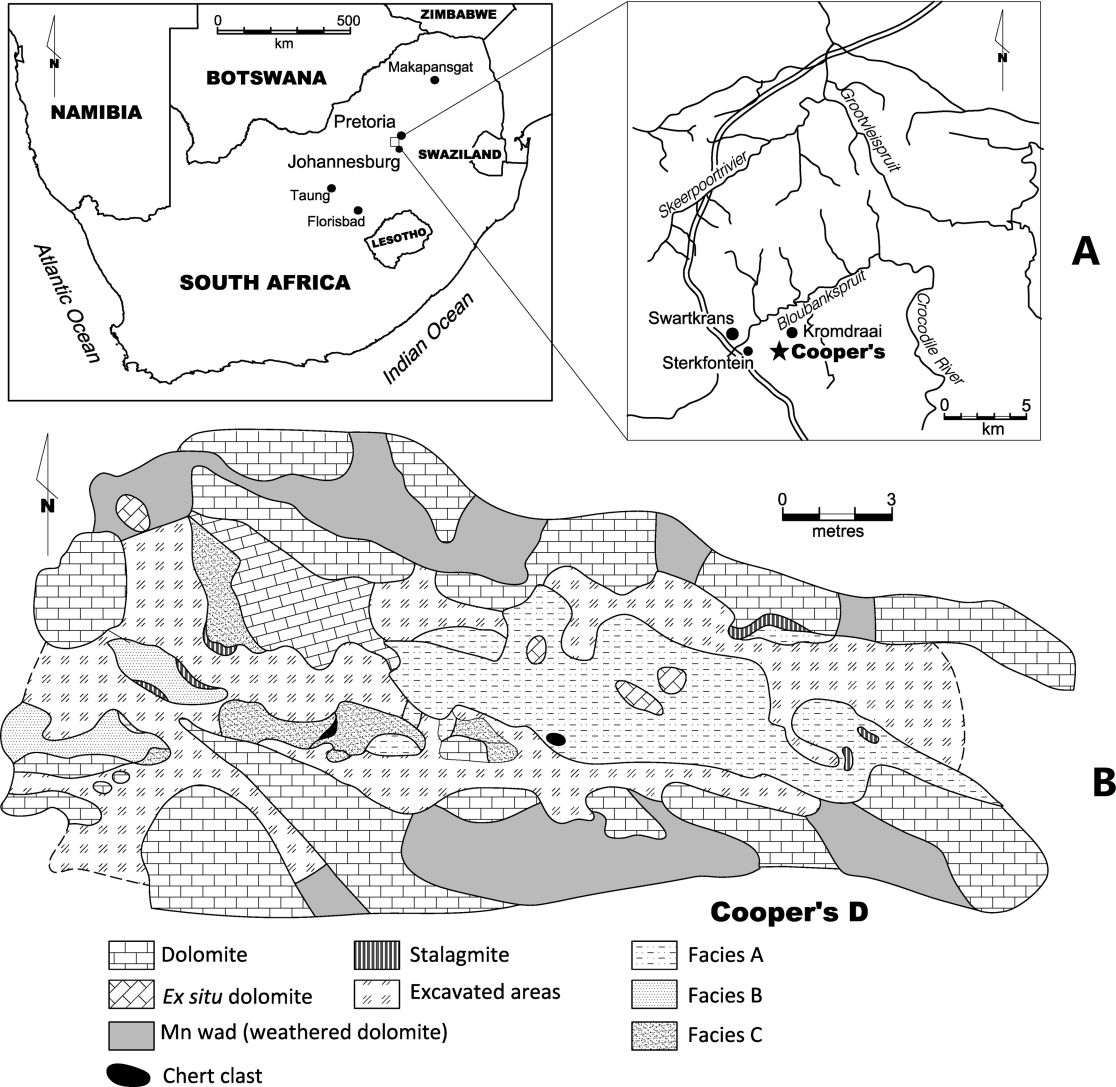
(A) Location of Cooper’s D in South Africa, and (B) site plan of the site. The location of Cooper’s D in the Gauteng province of South Africa. The site map is indicated

Cooper’s Cave is located in the Gauteng province of South Africa. The site is over 250 m^2^ in size and contains three distinct localities, Cooper’s A, B, and D all located in the Monte Cristo Formation (Malmani Subgroup, Transvaal Supergroup) (Fig. 1; see De Ruiter et al., 2009). The most fauna from Cooper’s Cave are from the D component, including the specimens described in this paper. Cooper’s D represents a de-roofed cave. Three facies are recognised at Cooper’s D (Facies A, B and C). Previous uranium-lead (U-Pb) analyses date Cooper’s D to between 1.5 and 1.4 million years ago (De Ruiter et al., 2009). Recently refined uranium-lead dates for Cooper’s D suggest a 1.38 ± 0.11 Ma date for the site (Pickering et al., 2019). Remains of *Paranthropus robustus* are present at Cooper’s D. This taxon has only been recovered at Swartkrans, Kromdraai, Sterkfontein, Gondolin and Drimolen in the Bloubank Valley and lived between 2 and 1.2 million years ago in South Africa (De Ruiter, Sponheimer & Lee-Thorp, 2008; Steininger, Berger & Kuhn, 2008).

The animal remains from Cooper’s D have been well studied. Berger et al. (2003) and De Ruiter et al. (2009) were the first to produce a preliminary list of taxa present in the faunal assemblage from Cooper’s D, and these include primates, carnivores, hyraxes, perissodactyls, artiodactyls, rodents and lagomorphs. Val, Taru & Steininger (2014) studied the primate remains from Cooper’s D, and argued that at least some primates, including *P. robustus*, were hunted and killed by leopards and hyenas. However, given the abundance of juvenile and sub-adult primates, the geomorphology of the cave, and the low impact of carnivore modification, Cooper’s D likely represented a locality that was occupied by large-bodied cercopithecids who died a natural death in the cave (Val, Taru & Steininger, 2014). Cooper’s D also yielded primate remains of *Theropithecus*, *Papio*, and a large papionion that may belong to *Gorgopithecus* (DeSilva, Steininger & Patel, 2013; Folinsbee & Reisz, 2013). O’Regan & Steininger (2017) studied the felid sample from Cooper’s D. This sample indicates the presence of four large felid genera, namely *Dinofelis*, *Megantereon*, *Panthera* and *Acinonyx*. This sample may mark the first appearance of the modern cheetah in Africa, as well the first occurrence of the East African species *Dinofelis cf. aronoki* in southern Africa. Moreover, the site also yielded remains of two mustelids, *Propoecilogale bolti* and *Mellivora capensis*, as well as a viverrid, *Civettictis cf. civetta* (O’Regan, Cohen & Steininger, 2013). The Herpestidae from Cooper’s D were collected by brown hyena (Cohen, O’Regan & Steininger, 2019). The Hyaenidae from Cooper’s D include *Chasmaporthetes nitidula*, *Crocuta ultra*, *Parahyaena brunnea*, *Hyaena hyaena* and *cf. Proteles* sp. (Kuhn, Werdelin & Steininger, 2017). The preliminary studies indicate the presence of the extant *Equus quagga* and extinct *Equus capensis* at Cooper’s D, (Berger et al., 2003; De Ruiter et al., 2009). As these Equidae specimens have not been thoroughly considered from the site, they are described for this paper, and their taphonomic and palaeoecological implications discussed.

### Brief Overview of the Taxonomic Status of Equidae

The taxonomic status of the Equidae in southern Africa has been debated for several decades. Morphometric analyses suggest that the plains zebra (or Burchell’s zebra) *Equus burchellii* and the extinct quagga *E. quagga* are distinct species (e.g., Thackeray, 1988; Klein & Cruz-Uribe, 1999). However, the evidence from cranial morphology, body stripes and molecular data consider *E. quagga* as a subspecies of the plains zebra (e.g., Lowenstein & Ryder, 1985; Rau, 1986; but see Leonard et al., 2005). It is now widely accepted that the two taxa are conspecific with the older name of *E. quagga* retained (Bronner et al., 2003; Skinner & Chimimba, 2005). There are three extant species of zebra in sub-Saharan Africa. They are Grévy’s zebra *Equus grevyi* from East Africa, the mountain zebra *Equus zebra* from the western and southern parts of southern Africa and south-western Angola, and *E. quagga* from the north-eastern parts of southern Africa west to Angola (Bronner et al., 2003; Skinner & Chimimba, 2005).

Early in the 21st century, Broom (1909) described a large extinct Equid from South Africa, which he named *Equus capensis*. This taxon, often referred to as the extinct Cape zebra had shorter limbs, was more heavily built than extant horses with a larger cranium (Brain, 1981). Poor understanding of individual variation in teeth morphology and the effects of wear on teeth resulted in a flurry of different extinct zebras described in South Africa (Wells, 1959). However, these are all now considered synonyms of *Equus capensis* (Churcher & Richardson, 1978).

The taxonomic status of *Equus capensis* remains controversial (e.g., Peters et al., 1994), but we retained the taxon following Bernor et al. (2010: 705). Some authors (e.g., Cooke, 1950; Churcher, 1986; Churcher, 2006) suggested that *Equus capensis* is closely related to the extinct *Equus oldowayensis* from East Africa, and its immediate descendant, Grévy’s zebra (*Equus grevyi*). *Equus grevyi* has a large head with short legs (Churcher & Richardson, 1978). According to this view, *Equus capensis* still exists as Grévy’s zebra in Eastern Africa today (Churcher, 2006). Recent studies on the DNA from *Equus capensis* indicate they are closer related to *Equus quagga* (Orlando et al., 2009). However, extensive osteological comparisons are still lacking between these forms (Peters et al., 1994:24). Often, in practice, Equid specimens of adult individuals that are larger than *Equuss quagga* are regarded as belonging to *Equus capensis* (e.g., Brink, 1987; Peters et al., 1994; Stynder, 1997; Badenhorst et al., 2011; Adams et al., 2016).

*Equus capensis* had a wide distribution in southern and eastern Africa, and existed from the Late Pliocene to the Early Holocene (*ca.* 3.6 mya–10,000 ka). Stable isotopes indicate that, on average *Equus capensis* were grazers (Lee-Thorp & Beaumont, 1995; Codron et al., 2008) of coarse grasses like modern plains zebras (Hofmann & Stewart, 1972; Stynder, 1997). In the interior of South Africa, *Equus quagga* and *Equus capensis* occur concurrently in many Pleistocene deposits (Churcher & Richardson, 1978). *Equus quagga* appeared about one million years ago in southern Africa, although they occurred in Africa from the Late Pliocene–Early Pleistocene (Bernor et al., 2010).

Another species of extinct zebra existed in southern Africa, formerly called *Hipparion lybicum steytleri* (Churcher & Richardson, 1978). Bernor et al. (2010) refers to the hipparion material from Ethiopia, Tanzania, Kenya and South Africa as *Eurygnathohippus cornelianus*, which existed between 2.5 and .05 million years ago. Hipparions have been identified at localities such as Swartkrans Member 1 and Kromdraai A (Brain, 1981). This three-toed zebra had a slightly-built, and appreciably smaller than *Equus quagga* (Brain, 1981) with distinct dental morphology (Churcher & Richardson, 1978; Brain, 1981; MacFadden, 1992; Churcher, 2000; Churcher & Watson, 2004; Churcher, 2006).

## Methods

All the available Equidae material from Cooper’s D was considered for this paper. Specimens were identified using the collections housed at the University of the Witwatersrand (modern BP and fossil Swartkrans SK collections) and the Ditsong National Museum of Natural History (AZ collection) coupled with published descriptions (Broom, 1909; Cooke, 1950; Churcher, 1974; Churcher & Richardson, 1978; Brain, 1981; Churcher, 2000; Churcher & Watson, 2004; Churcher, 2006). Following common practice (e.g., Brink, 1987; Peters et al., 1994; Stynder, 1997; Badenhorst et al., 2011; Adams et al., 2016), specimens larger than the extant *Equus quagga* were identified as the extinct *Equus capensis*. The specimens are quantified using the Number of Identified Specimens (NISP) and the Minimum Number of Individuals (MNI). Detail about each identified specimen is listed in Data S1.

All measurements for postcranial material follow Von den Driesch (1976). Comparative measurements are included from specimens housed in the collections at the University of the Witwatersrand and the Ditsong National Museum of Natural History. Almost no comparative measurements are available for Equidae from sites in the Bloubank Valley (but see Churcher & Watson, 2004). It is well beyond the scope of this paper to include such a comprehensive osteometrical study. However, we included measurements of *Equus quagga* and *Equus capensis* where such data is available for comparative purposes. All measurements are in millimetres to accuracy of 0.1 mm, and when an estimated measurement was taken, it is indicated as ‘est’, following common practice in osteometrical research (e.g., Von den Driesch, 1976). For the II and IV metapodia of Equidae, an anterior-posterior length was taken of the proximal part of the element, as well as a medial-lateral breadth measurement. We calculate the proportion of Equidae to large and very large Bovidae (Bov III’s and IV’s, following Brain (1974) in samples from the Bloubank Valley where remains of *P. robustus* have been discovered. We selected large and very large Bovidae, as these ungulates are similar in size to Equidae, and may have been prey to similar predators.

## Results

**Figure 2:**
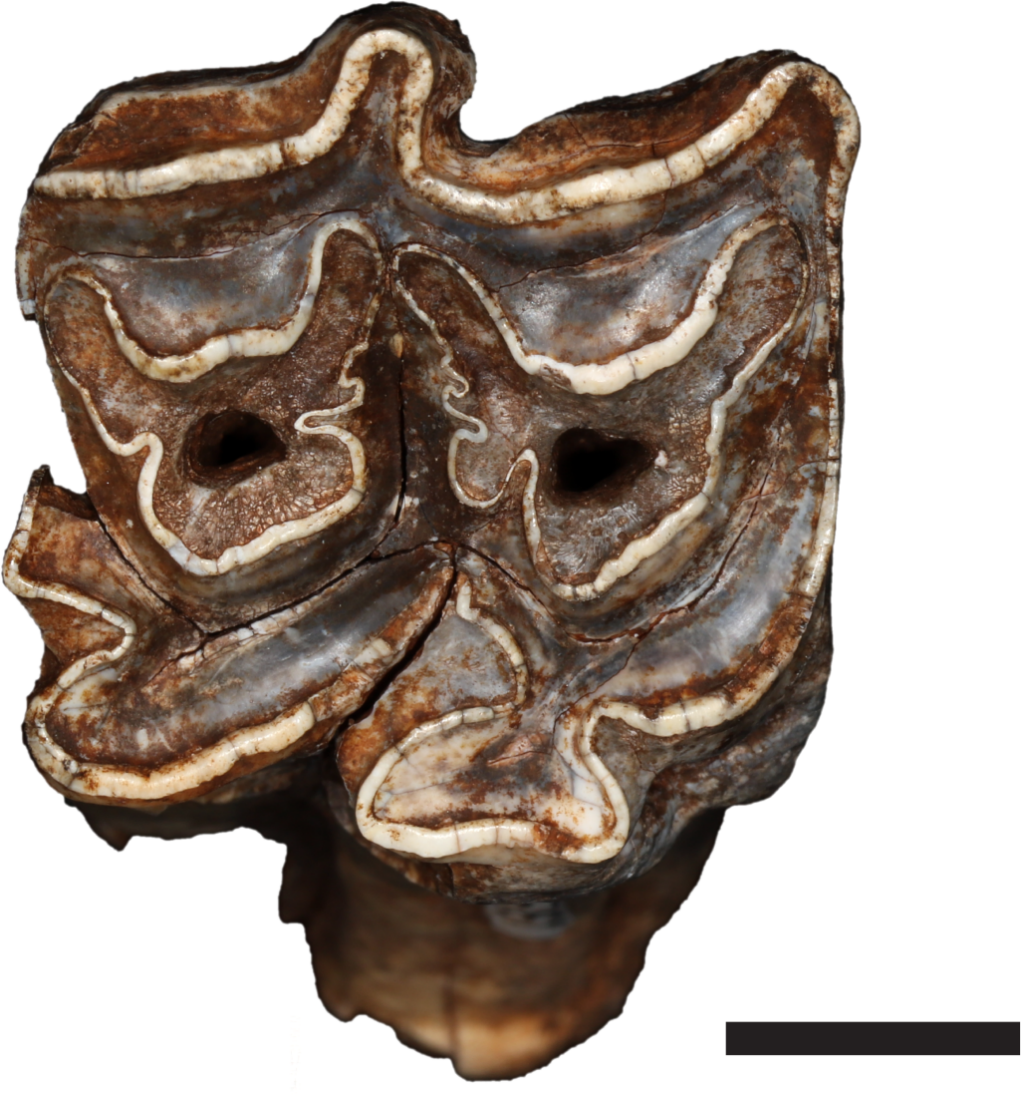
Example of teeth assigned to *Equus capensis* (Specimen CD 5881, Upper M1). Note that the protocone is not isolated as is the case in hipparions. Photo credit: Ashley Kruger.

**Figure 3:**
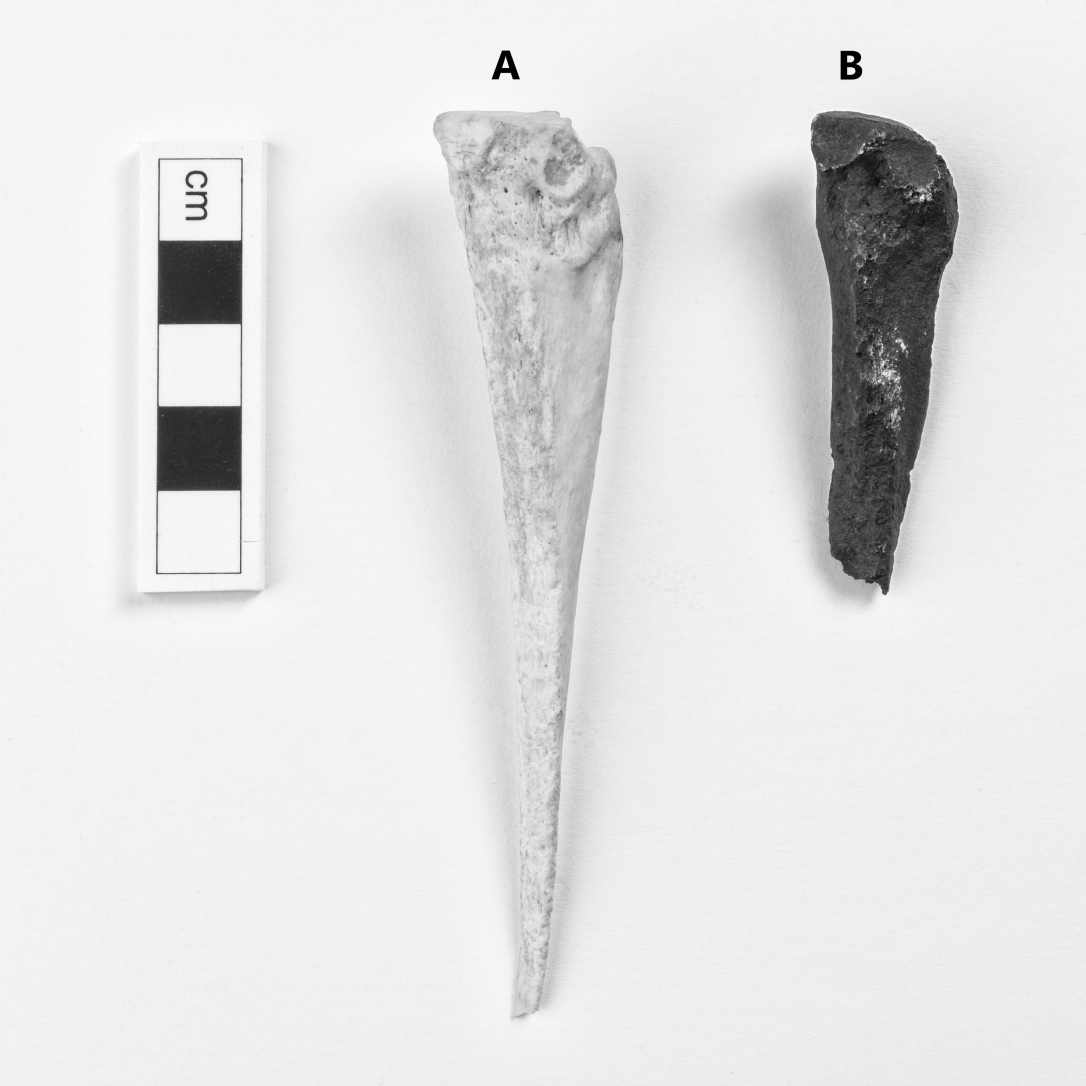
(A) a right metacarpal IV of *Equus quagga* (AZ 1131). B: CD 24345, a right metacarpal IV, which is likely from *Eurygnathohippus cornelianus* (see Table 2). Photo credit: Brett Eloff.

The extinct *Equus capensis* and *Eurygnathohippus cf. cornelianus* were identified from Cooper’s D (Table 1). Many specimens from Cooper’s D retained sufficient morphological features to allow identification to species level. *Equus capensis* is represented by at least one adult and one juvenile individual in the sample. No taphonomic modifications were noted on the material. None of the teeth from Cooper’s D could be assigned to hipparion (Fig. 2). However, one postcranial specimen is likely from a hipparion (Fig. 3). It is a right metacarpal IV, which is smaller in size than the average *Equus quagga* (Table 2) and larger than a modern donkey. As a result, we postulate that this specimen is a hipparion (*Eurygnathohippus cf. cornelianus*). Teeth and post-crania of *Equus capensis* are larger in size than *Equus quagga* (Tables 3 and 4). Specimen CD 991 is proximal portion of a left IV metatarsal, and specimen CD 3508 is a proximal portion of a left metatarsal II. Both are identified as *Equus capensis* due to their large size (Table 5). Using NISP, most samples contain between 4 and 15% Equidae (Swartkrans Members 1, 2 and 3, Drimolen and Sterkfontein Member 5). At Cooper’s D, Equidae are also poorly represented (3% based on MNI). Gondolin 1 and 2 has the highest representation of Equidae of 30%. Kromdraai contains no Equidae remains (Table 6).

**Table 1:** The Equidae identified in the Cooper’s Cave assemblage.

**Taxon**	**NISP**	**MNI**
*Equus capensis*	13	2(1 adult,1 juvenile)
*cf. Equus capensis*	3	–
*Eurygnathohippus cornelianus*	1	1
Equidae indeterminate	18	–
**Total**	**42**	

## Discussion

*Equus capensis* was identified based on the large size of the specimens compared to the smaller extant *Equus quagga*. The use of size differences to distinguish Pleistocene and extant species remains controversial (Watson & Plug, 1995), also in the case of *Equus capensis* (Peters et al., 1994). However, extensive morphological comparisons are lacking to determine if *Equus capensis* is related to an extant species of zebra (Peters et al., 1994). *Equus capensis* appeared some two million years ago in South Africa, and existed until about 10 000 years ago. In contrast, the plains zebra only appeared about a million years ago in eastern and southern Africa, and still exists today (summary in Bernor et al. (2010):702). This suggests that the two taxa had different evolutionary trajectories as two separate species or subspecies. However, more research is required to investigate the relationship between them. Assuming *Equus capensis* is a distinct species, it is present at Cooper’s D. The deposit predates the appearance of *Equus quagga* in the Bloubank Valley by millennia. *Equus capensis* occurs at sites in the Bloubank Valley like Swartkrans (Members 1–3) (De Ruiter, 2003), Gladysvale (GVED) (Lacruz et al., 2003), Sterkfontein Member 4, Kromdraai A (Brain, 1981) and Garage Ravine at Bolt’s Farm (Badenhorst et al., 2011).

**Table 2:** Comparison between metacarpal IV Cooper’s D *Eurygnathohippus cf. cornelianus* and modern Equidae. For some specimens, the sex is unknown.

**Taxa**	**Specimen Number**	**Sex**	**Length**	**Breadth**
*Eurygnathohippus cf. cornelianus*	CD 24 345	–	18.65	14.14
*Equus quagga*	AZ 3252	–	22.45	16.57
*Equus quagga*	AZ 1283	♀	19.79 (est)	–
*Equus quagga*	AZ 1131	–	23.81	16.0
*Equus caballus*	AZ 585	♀	22.52 (est)	18.26 (est)
*Equus africanus asinus*	AZ 423	–	15.78	12.0

**Table 3:** Comparisons of teeth measurements of *Equus capensis* from Cooper’s D and *Equus quagga*. The measurements indicate *Equus capensis* is consistently larger than *Equus quagga*.

**Taxa**	**Specimen Number**	**Side**	**Mesiodistal Length**	**Buccolingual Breadth**
**Upper M1**				
*Equus capensis*	CD 5881	R	30.28 (est)	30.47
*Equus quagga*	BP/4/147	R	23.46 (est)	26.38
**Upper M3**				
*Equus capensis*	CD 16973	R	31.06	25.98
*Equus quagga*	BP/4/147	R	21.67 (est)	20.89
**Indeterminate Upper Molar**				
*Equus capensis*	CD 9070	R	36.19	26.99
*Equus capensis*	SK 41515	–	31.80	22.46
**Upper Deciduous P3 or P4**				
*Equus capensis*	CA 1159	R	38.55	25.84 (est)
*Equus capensis*	CD 9293	R	36.20	26.40
*Equus capensis*	SK 28881	R	39.1	28.2
**Indeterminate Lower Molar**				
*Equus capensis*	CD 992	–	27.10	–
*Equus quagga* (M1)	BP/4/147	–	22.25	–
*Equus quagga* (M2)	BP/4/147	–	22.60	–
**Lower M3**				
*Equus capensis*	CD 11067	L	40.65 (est)	18.48 (est)
*Equus quagga*	BP/4/147	L	31.20 (est)	15.22 (est)

**Table 4:** Comparisons of measurements of post-crania of *Equus capensis* from Cooper’s D with other Equidae. *Equus capensis* is consistently larger than *Equus quagga* , and similar in size to the modern horse *Equus caballus*.

**Taxa**	**Specimen Number**	**Sex**	**Side**	**Measurements**
**Astragalus**				
*Equus capensis*	CD 6747	–	Left	GH: 61.58, LmT: 59.68, GB: 59.12, BFd: 46.26
*Equus capensis*	SK 6288	–	Right	GH: 63.04, LmT: 63.23
*Equus quagga*	AZ 1424	♀	Left	GH: 54.56, LmT: 54.80, GB: 59.15, BFp: 46.46
**Scapula**				
*Equus capensis*	CD 6061	–	Left	GLP: 103.00, SLC: 72.15, LG: 65.31, BG: 56.11
*Equus quagga*	AZ 1424	♀	Left	GLP: 77.78, SLC: 51.01, LG: 48.10, BG: 42.60
*Equus quagga*	BP/4/911	–	–	GLP: 75.73, SLC: 50.30, LG: 49.72, BG: 38.54
*Equus caballus*	BP/4/929	–	–	GLP: 100.33 (est), SLC: 69.34, LG: 57.19, BG: 49.06
**Femur**				
*Equus capensis*	CD 10416	–	Left	DC: 56.94
*Equus quagga*	AZ 1424	♀	Left	DC: 48.71

**Notes.**

Astragalus GH greatest height LmT length of the medial part of the trochlea tali GB greatest breadth BFd breadth of the facies articularis distalis Scapula GLP greatest length of the processus articularis SLC smallest length of the collum scapulae LG length of the glenoid cavity BG breadth of the glenoid cavity Femur DC greatest depth of the caput femoris (Von den Driesch, 1976)

**Table 5:** Comparisons of measurements of metatarsals II and IV of *Equus capensis* from Cooper’s D and *Equus quagga*. The measurements indicate *Equus capensis* is consistently larger than *Equus quagga*.

**Taxa**	**Specimen Number**	**Sex**	**Length of Proximal Articulation**	**Breadth of Proximal Articulation**
**Metatarsal IV**				
*Equus capensis*	CD 991	–	32.77	22.26 (est)
*Equus quagga*	AZ 1424	–	25.81	19.40
*Equus quagga*	AZ 1131	–	27.61	20.30
*Equus quagga*	AZ 1132	♂	27.05	18.94
*Equus quagga*	AZ 3252	–	26.04	20.08
*Equus quagga*	AZ 1283	♀	27.16	17.84
**Metatarsal II**				
*Equus capensis*	CD 3508	–	23.13	16.09
*Equus quagga*	AZ 1424	–	17.85	11.94
*Equus quagga*	AZ 1131	–	17.54	11.71
*Equus quagga*	AZ 1132	♂	18.18	11.68
*Equus quagga*	AZ 3252	–	16.77	11.90
*Equus quagga*	AZ 1283	♀	18.40	11.83

**Table 6:** Proportions of Equidae and large and very large Bovidae (Bovidae III and IV, see Brain (1974) representation in samples containing *Paranthropus robustus*. The dates are from Herries, Curnoe & Adams (2009).

Site and samples	**Relative age (mya)**	**Equidae (NISP/MNI)**	**Bovidae III and IV (NISP/MNI)**	**% Equidae NISP (%MNI)**	**Reference**
Swartkrans Member 1	2	27/11	594/82	4 (12)	Brain, 1981: 322–323
Swartkrans Member 1	2	30/4	418/12	7 (25)	Watson, 2004: 40
Kromdraai B	2.11–1.65	-/-	66/12	0 (0)	Brain, 1981: 339–340
Kromdraai Member 2	2.11–1.65	-/-	84/-	0 (0)	Fourvel et al., 2016: 83
Gondolin 1 and 2	1.78	19/2	45/8	30 (20)	Adams et al., 2007: 2533
Sterkfontein Member 5	1.78–0.82	23/3	157/15	13 (17)	Brain, 1981: 317
Drimolen	2–1.5	3/1	64/23	4 (4)	Adams et al., 2016
Cooper’s D	1.5–1.4	-/2	-/67	- (3)	De Ruiter et al., 2009: 506. This study
Swartkrans Member 2	1.65–1.07	60/16	335/43	15 (27)	Brain, 1981: 328–329
Swartkrans Member 2	1.65–1.07	22/4	268/15	8 (21)	Watson, 2004: 40
Swartkrans Member 3	1.04–0.62	74/7	892/34	7 (17)	Watson, 2004: 40

The extant *Equus quagga* is present in Africa since 2.33 million years ago (Geraads, Raynal & Eisenmann, 2004; Adams et al., 2016). Remains have been found at sites in the Bloubank Valley such as Swartkrans (Member 3) (De Ruiter, 2003), Drimolen Main Quarry (Adams et al., 2016), Gladysvale (GVED) (Lacruz et al., 2003), Sterkfontein (Members 5 and 6), Swartkrans Channel Fill and Kromdraai A (Brain, 1981). A previous, preliminary analysis of the Equidae reported *Equus quagga* from Cooper’s D (Berger et al., 2003; De Ruiter et al., 2009).

The small size of the specimen, comparable to modern donkey, suggest that the specimen originally designated as *Equus quagga* from Cooper’s D is actually *Eurygnathohippus cf. cornelianus*. The latter species is only recognised from teeth and lower leg bones, and no morphological differences necessarily exist to separate the smaller *Eurygnathohippus cornelianus* from *Equus quagga*. *Eurygnathohippus cornelianus* is not abundant in fossil deposits in the interior of South Africa, but specimens have been found at Cornelia-Uitzoek (Brink et al., 2012), Makapansgat Member 3 (Reed, 1996), Kromdraai A (Churcher, 1970), Swartkrans Members 1–3 (Churcher & Watson, 2004), Sterkfontein Member 4 (Reynolds & Kibii, 2011), Gondolin GD 2 (Adams, 2006) and Drimolen Makondo Infill (Rovinsky et al., 2015). *Eurygnathohippus cornelianus* occur mainly in Pliocene deposits in South Africa, but they persist to the Early Pleistocene as is evident from Cornelia-Uitzoek, which dates to between 1.07 and 0.99 million years ago (Brink et al., 2012; Rovinsky et al., 2015).

Previous research at Cooper’s D suggests brown hyena and leopard involvement in the accumulation of the deposits (De Ruiter et al., 2009; Cohen, O’Regan & Steininger, 2019). At sites like Swartkrans, Sterkfontein, Kromdraai and Minnaar’s Cave, the extinct sabre-toothed cat, *Dinofelis* could have preyed on *Equus capensis* (Lesnik & Thackeray, 2006) although other predators could also have been involved. The main predators of extant Grévy and plains zebras are lions, leopards, spotted hyenas and hunting dogs (Smuts, 1979; Grubb, 1981; Churcher, 1993). Of these predators, lions and hunting dogs do not make use of shelters (e.g., Pitman et al., 2013) although carnivores such as hyenas could scavenge their kills and bring bones into shelters. The low to near absence of Equid remains at sites in the Bloubank Valley may reflect this. Small sample sizes aside (Rovinsky et al., 2015), Equidae may have also been absent from some portions of the Bloubank Valley and larger region for some times of the year (Mills & Shenk, 1992). In addition, while Equids may have been regularly preyed upon, not all attempts to hunt them would have been successful. Plains and Grévy’s zebras are formidable prey, being able to kick and bite to defend themselves and run at high speeds (overviews in Grubb, 1981; Churcher, 1993).

Like the plains and Grevy’s zebra (Rautenbach, 1982; Churcher, 1993; Skinner & Chimimba, 2005), *Equus capensis* (Lee-Thorp & Beaumont, 1995; Codron et al., 2008) and *Eurygnathohippus* were grazers (Franz-Odendaal, Kaiser & Bernor, 2003; White et al., 2009; Cerling et al., 2015). The plains zebra must drink water daily, and is always in close proximity to water (Rautenbach, 1982). Grévy’s zebra require less water than the plain zebra. While they prefer to drink daily, they can go without water for three days (Churcher, 1993). The source of water was presumably the palaeo-Bloubank River (De Ruiter et al., 2009). The large mammalian fauna from Cooper’s D suggest a mosaic environment with grassland, woodland and water components (De Ruiter et al., 2009; Steininger, 2011; Cohen, O’Regan & Steininger, 2019).

## Conclusion

The Equidae sample from Cooper’s D contains two extinct species, *Equus capensis and Eurygnathohippus cf. cornelianus.* While a previous preliminary report indicate the presence of the extant *Equus quagga*, we do not consider this identification to be correct. Instead, the small size of the specimen, and the deposit predating the arrival of extant *Equus quagga* in South Africa, supports this notion. A variety of carnivores preyed on Equidae including hyenas, leopards and lions and likely contributed bone remains to the deposits, directly or indirectly. The presence of the two extinct species supports the presence of grassland, woodland and water components around Cooper’s D at 1.38 ± 0.11 Ma.

##  Supplemental Information

10.7717/peerj.6909/supp-1 Data S1 Description of fauna The different specimens discussed in this article.

## Additional Information and Declarations

### Data Availability

The following information was supplied regarding data availability: Detail for each identified specimen is listed in Data S1.
