# Supplementary material for: The Equidae from Cooper’s D, an early Pleistocene fossil locality in Gauteng, South Africa
Source: PeerJ. 2019 May 15;7:e6909. doi: 10.7717/peerj.6909 (PMC6525595; doi:10.7717/peerj.6909)
Supplement: Data S1 — The different specimens discussed in this article. [file peerj-07-6909-s001.docx]

**Appendix A: Systematic Palaeontology**

Order PERISSODACTYLA Owen, 1848

Family EQUIDAE Gray, 1821

*Equus capensis*

| **Specimen** | **Element** | **Side** | **Description and Additional Measurements** |
| --- | --- | --- | --- |
| CD 5881 | Upper 1^st^ molar | R | Roots absent, and part of mesial aspects of the enamel also absent. Some wear. Adult. The crown height on the buccal – mesial side is 77.76 mm |
| CD 16973 | Upper 3^rd^ molar | R | Roots absent. Some wear. Adult. The hypocone – lingual crown height is 57.74 mm |
| CD 11067 | Lower 3^rd^ molar | L | Mesial root absent. Some wear. Adult. The mid-lobe lingual crown height is 51.97 mm |
| CD 992 | Lower molar fragment, likely the 1^st^ or 2^nd^ molar | - | Part of enamel surface present, with roots absent. Some wear. Adult |
| CD 993 | Upper 1^st^ molar | L | The roots are absent, as well as part of the crown. Little wear on tooth. Young adult |
| CD 9070 | Upper molar | R | Most of the crown is present, but the roots are absent. The enamel surfaces are not fully developed yet. Young individual. The tooth is large |
| CD 9293 | Upper deciduous 3^rd^ or 4^th^ premolar | R | Tooth largely intact with roots absent. Some wear. Young individual. The protocone crown height is 9.21 mm |
| CA 1159 | Upper deciduous 3^rd^ or 4^th^ premolar | R | Almost no wear. Young individual. Tooth complete, but the roots are absent |
| CD 991 | Metatarsal IV | L | Complete proximal articulation and proximal shaft |
| CD 6747 | Astragalus | L | Complete |
| CD 6061 | Scapula | L | Tuber scapulae, anterior and posterior glenoid cavity, and portion of the blade present |
| CD 10 416 | Femur | L | Proximal femur with head, trochanter fossa and neck, and part of the trochanter minor. Fused. Adult |
| CD 3508 | Metatarsal II | L | Complete proximal articulation and proximal shaft |

*cf. Equus capensis*

| **Specimen** | **Element** | **Side** | **Description and Additional Measurements** |
| --- | --- | --- | --- |
| CD 15673 | 1^st^ upper incisor | L | Near complete, 1^st^ upper incisor. Only part of the root is absent. The maximum width of the enamel is 19.96 mm, and that of *Equus quagga* BP/4/147 is 14.82 mm |
| CD 10424 | Tibia | R | Distal articulation of tibia and distal shaft. Part of medial and lateral articulations absent. Fused. Adult |
| CD 9375: | Mandible | L | Medial condyle and neck of mandible |

*Eurygnathohippus cf. cornelianus*

| **Specimen** | **Element** | **Side** | **Description** |
| --- | --- | --- | --- |
| CD 24345 | Metacarpal IV | R | Complete proximal articulation and proximal shaft |

*Equidae indeterminate*

| **Specimen** | **Element** | **Side** | **Description** |
| --- | --- | --- | --- |
| CA 20197 | Incisor | - | Deciduous incisor fragment of a young individual |
| CD 10 662: | Incisor | - | Incisor fragment |
| CA 19319 | Lower molar | R | Molar fragment of a young individual |
| CD 13321 | Upper I3 | L | Very little wear. Young adult |
| CD 13484 | Incisor | - | Incisor fragment |
| CD 13519 | Upper 2^nd^ incisor | L | Incisor fragment. Some wear. Adult |
| CD 22835 | Deciduous incisor | L | Incisor fragment. Young individual |
| CD 13326 | Upper incisor | R | Incisor fragment. Some wear. Adult |
| CD 13495 | Lower incisor | L | Incisor fragment. Some wear. Adult |
| CD 21933 | Un-erupted tooth | - | Un-erupted tooth fragment. Young individual |
| CD 1211 | Lower incisor | - | Incisor fragment. Worn. Adult. As lower incisors tend to wear down quicker than upper incisors, this specimen is not necessarily from an aged individual |
| CD 989 | Lower tooth | - | Tooth fragment |
| CD 6750 | Tarsal | L | Tarsal fragment |
| CD 1485 | Tarsal | - | Tarsal fragment |
| CD 17387 | Humerus | R | Deltoid tuberosity of humerus |
| CD 2415 | Metapodial | - | Unfused distal articulation. Young individual |
| CD 7974 | 2^nd^ phalanx | - | Distal articulation and shaft present. Young individual |
| CD 10 425: | Radius | R | Distal shaft of radius |
